# New Insights and Current Approaches in Cardiac Hypertrophy Cell Culture, Tissue Engineering Models, and Novel Pathways Involving Non-Coding RNA

**DOI:** 10.3389/fphar.2020.01314

**Published:** 2020-08-21

**Authors:** Nina Kastner, Katrin Zlabinger, Andreas Spannbauer, Denise Traxler, Julia Mester-Tonczar, Ena Hašimbegović, Mariann Gyöngyösi

**Affiliations:** Department of Cardiology, Medical University of Vienna, Vienna, Austria

**Keywords:** cardiac hypertrophy, disease modeling, hypertrophy induction, non-coding RNA, tissue engineering

## Abstract

Cardiac hypertrophy is an ongoing clinical challenge, as risk factors such as obesity, smoking and increasing age become more widespread, which lead to an increasing prevalence of developing hypertrophy. Pathological hypertrophy is a maladaptive response to stress conditions, such as pressure overload, and involve a number of changes in cellular mechanisms, gene expression and pathway regulations. Although several important pathways involved in the remodeling and hypertrophy process have been identified, further research is needed to achieve a better understanding and explore new and better treatment options. More recently discovered pathways showed the involvement of several non-coding RNAs, including micro RNAs (miRNAs), long non-coding RNAs (lncRNAs), and circular RNAs (circRNAs), which either promote or inhibit the remodeling process and pose a possible target for novel therapy approaches. *In vitro* modeling serves as a vital tool for this further pathway analysis and treatment testing and has vastly improved over the recent years, providing a less costly and labor-intensive alternative to *in vivo* animal models.

## Introduction

Left ventricular hypertrophy is a consequence of hypertension in up to 30% of patients (Cramariuc and Gerdts, 2016). Due to widespread risk factors such as obesity and smoking, the prevalence of hypertension and subsequent myocardial hypertrophy is rising, which poses a significant public health burden in an aging population (Benjamin et al., 2018). This development combined with the relative paucity of direct treatment options for cardiac hypertrophy makes continued research and the identification of novel therapeutic target molecules absolutely vital. Cardiac hypertrophy is an adaptive process which develops in response to physiological but also pathological processes, leading to heart muscle and cell hypertrophic growth with increased rigidity of the heart structures, and impaired diastolic function leading to heart failure with preserved ejection fraction (HFpEF) (Loonat et al., 2019; Zhao et al., 2020). Hypertrophic growth involves cardiomyocyte enlargement rather than division, as adult cardiomyocytes have lost the ability to divide (Porrello et al., 2013). Pathological hypertrophy leads to the loss of functional cardiomyocytes (Nakamura and Sadoshima, 2018) and can subsequently progress to heart failure with reduced ejection fraction (HFrEF) (Tham et al., 2015). The main focus in hypertrophy research lies in the investigation of signaling pathways, gene expression analysis, and production of certain proteins and transcription factors, which influence or are responsible for the remodeling process. Many pathways have been identified to be involved in the development of cardiac hypertrophy including calcineurin/nuclear factor of activated T cells (NFAT), mitogen-activated protein kinase ERK, small guanosine triphosphate (GTP)-binding proteins (Ras, Rho), PKC, transcriptional regulation, cell surface level control, miRNA, and many more (Stansfield et al., 2014). Hypertrophic signaling pathways are initiated through mechanical stimulation such as pressure overload and neurohumoral mechanisms including the release of signaling factors such as growth factors, hormones, cytokines, and chemokines (Heineke and Molkentin, 2006).

Non-coding RNAs are RNAs that do not code for a protein and are not translated, however they have been shown to interfere with and regulate numerous physiological and pathophysiological pathways. There are different classes of non-coding RNAs, include microRNAs (miRNAs), long non-coding RNAs (lncRNAs) and circular RNAs (circRNAs), depending on their structure, length and function (Jaé and Dimmeler, 2020). MiRNAs are small RNAs with a length of approximately 22 nucleotides, which can inhibit mRNA translation and signal mRNA degradation (O’Brien et al., 2018). LncRNAs comprise of over 200 nucleotides and they can induce structural changes in DNA and chromatin, therefore regulating gene expression, and bind miRNAs thereby inhibiting their function (Dhanoa et al., 2018). Similarly, circRNAs also act as a sponge for miRNAs, however their structural properties are different to other non-coding RNAs, as they are circularized and are more robust resistant to RNAses (Yu and Kuo, 2019).

The purpose of this mini-review is to summarize the developments in myocardial hypertrophy models, new pathways, tissue engineering and non-coding RNAs relevant in myocardial hypertrophy induction and progression.

## Cardiac Hypertrophy Cell Culture and Tissue Engineering Models

Cell culture models are essential for the investigation of pathological and physiological pathways in cardiac diseases. In contrast, *in vivo* animal models more closely simulate human physiological and pathological conditions. In cardiac research the most popular animal models are rodents such as rats and mice (Zuppinger, 2019). However, they differ from human physiology in some key aspects, such as faster heart rates and stem cell phenotypes (Ginis et al., 2004; Jochmans-Lemoine et al., 2015). Several large animal models using pigs, sheep, or dogs have been established to more accurately simulate human pathophysiology and cardiac hypertrophy with HFpEF and HFrEF, but very high cost and labor intensity limit their use for specialized research questions (Spannbauer et al., 2019). Sophisticated *in vitro* models are therefore an important tool to elucidate new remodeling and hypertrophy pathways and identify molecules of interest before proceeding to costly translational models.

The most widely used technique is a 2D monolayer cell culture, which allowed the discovery of several important hypertrophy pathways and cellular mechanisms, but lacks the complexity of interactivity between cells and cell types with electrical and paracrine factors (Zuppinger, 2016). To achieve a more realistic *in vitro* model of cardiac hypertrophy where native *in vivo* niche conditions can be imitated, cell culture techniques have vastly improved in recent years, giving rise to 3D cultures and organoids (Dutta et al., 2017). Cardiac organoids have been developed and have steadily improved, however the optimal ratio of cardiomyocytes, fibroblasts and endothelial cells is the subject of ongoing debate (Nugraha et al., 2019). The most prominent techniques for producing cardiac organoids include the use of hydrogels such as collagen, cell sheet technology where cells are cultured and subsequently stacked in different layers and hanging drop culture (Dutta et al., 2017; Nugraha et al., 2019). Despite their many advantages, it is important to note that cardiac organoids in their current state are still far from the complexity of the organs they are meant to represent and are also unable to capture organism-wide processes like immune response or neurohumoral feedback mechanisms (Iakobachvili and Peters, 2017), which also limit their use in modeling of *in vivo* myocardial hypertrophy.

## Methods of Cardiac Hypertrophy Induction

*In vitro* cardiac hypertrophy can be induced by various methods such as chemical stimulation or mechanical stress induction. Mechanical stress induction is achieved by pressure overload *in vivo* and simulated by stretching of cardiomyocytes *in vitro*. On a cellular level, an activation of different oncogenes such as c-fos, c-myc, c-jun, and Egr-1, also called immediate-early genes, and activation of heat shock protein-70 are triggered and lead to subsequent upregulation of hypertrophy inducing genes ANP, BNP, and β-MHC (Rysä et al., 2018).

Pharmacological agents that are used for hypertrophy induction are for example phenylephrine (Jain et al., 2018), angiotensin II (Gelinas et al., 2018), noradrenaline (Guven, 2018), endothelin-1 (Zlabinger et al., 2019), and isoproterenol (Zhang et al., 2016). These agents interfere with a known pathway that is crucial in remodeling and subsequent cardiac hypertrophy. Angiotensin II was described to play a role in cardiac hypertrophy induction over 40 years ago, as it is part of the Renin-Angiotensin-Aldosterone-System. Endothelin-1 was discovered to have a higher protein and mRNA expression in dilated cardiomyopathy and influence matrix metalloproteinase 9 (Mmp9) expression levels (Hathaway et al., 2015). Further, a significant difference in mortality and pathogenesis has been reported in patients with a gene variation of the endothelin type A receptor gene (polymorphisms G231A and C1363T) (Herrmann et al., 2001; Telgmann et al., 2007). Noradrenaline is a stress induced neurotransmitter, which has been shown to be involved in cardiac hypertrophy and damage due to toxic effects on cardiomyocytes (Jain et al., 2015). Isoproterenol is a β-agonist and activates the Renin-Angiotensin-Aldosterone-System, thus inducing hypertrophic pathways (Leenen et al., 2001). All these pharmacologic agents induce hypertrophy by binding to specific G-coupled receptors on the cell membrane, which then induce diacylglycerol (DAG) and subsequently protein kinase C (PKC). PKC plays an important role in internal Ca^2+^ mobilization, which allows hypertrophic induction through NFAT or calmodulin-dependent kinase (CaMK) pathway interference (Heineke and Molkentin, 2006).

Induction of hypertrophy by all of these chemical agents show a similar modeling time of 24 h, which may be extended to 48 h depending on the used cell type (Zhang et al., 2016; Zlabinger et al., 2019; Zhao et al., 2020). The concentration needs to be optimized by testing different dilutions before the experiment, however the general used concentrations are 20 µM phenylephrine (Gelinas et al., 2018), 100 nM angiotensin II (Zlabinger et al., 2019), 10 μM isoproterenol (Zhang et al., 2016), and 10 nM endothelin-1 (Loonat et al., 2019).

## Cells Sources and Models for *In Vitro* Cardiac Hypertrophy Modeling

Establishing a cardiac hypertrophy model needs to incorporate several decisions about induction models, cell sources, pathways of interest and cell culture types. **Table 1** gives a broad overview about established approaches, while also mentioning the investigated pathway of the respective study. It is evident that a preferred choice of cell source are rat cardiomyocytes, which are mostly cultured in a 2D cell culture and the induction of hypertrophy is often performed chemically with pharmacological agents such as angiotensin II or isoproterenol. These approaches are based on availability of the cell source, as rat or also murine tissue for cell extraction is more easily accessible and animal care is cheaper to maintain than with larger animals. Further, chemical induction is a relatively easy method for hypertrophy induction compared to mechanical stress induction, which presumably is why it is often preferred.

**Table 1:** Overview over different approaches of hypertrophy induction methods and used cell sources in *in vitro* models and the investigated pathway of the study.

Cell source	Hypertrophy Model	Involved pathways	Reference
Neonatal rat ventricular myocytes (NRVM)	2D Cell culture, Chemical hypertrophy, Ang II, PE	AMPK pathway, O-GlcNAc signalling	(Gelinas et al., 2018)
Adult rat ventricular myocytes (ARVM)	2D Cell culture, Chemical hypertrophy, PE	AMPK pathway, O-GlcNAc signalling	(Gelinas et al., 2018)
Neonatal rat cardiomyocytes	2D Cell culture, Chemical hypertrophy, PE	Protein kinase D (PKD) knockdown, AKT/mTOR signaling pathway, autophagy	(Zhao et al., 2020)
Murine neonatal cardiomyocytes	Micro ridges vs 2D cell culture, Chemical hypertrophy, PE	Cardiomyocyte morphology on 3D surfaces – F-Actin, Myomesin, Actinin	(Jain et al., 2018)
H9c2 cardiomyocytes	2D Cell culture, Chemical hypertrophy, Iso	ANP, BNP, ROS, 3-nitrotyrosine and p67 (phox), MMP, (p)ERK1/2, (p)p38, (p)JNK	(Zhang et al., 2016)
Primary neonatal rat ventricular myocytes	2D Cell culture, Chemical hypertrophy, PE, ET1, Iso, Ang II	Myocyte area, protein-bound SRB fluorescence	(Loonat et al., 2019)
hiPSC from dermal fibroblasts edited with Crispr/Cas9	2D Cell culture/3D matrigel mattress, Mutational hypertrophy, heterozygous Ca-sensitizing TnT-I79N mutation	TnT-I79N protein levels, cytosolic Ca buffering and electrophysiology	(Wang et al., 2017)
Rat ventricular cardiomyocytes	2D Cell culture, Chemical hypertrophy, Ang II	GATA4 and miR-26a (also β-MHC and ANF)	(Liu et al., 2016)
Neonatal rat ventricular myocytes	2D Cell culture, Chemical hypertrophy, Ang II	β-catenin, NFATc3, WNT pathway	(Jiang et al., 2018)
H9c2 cardiomyocytes	2D Cell culture, Chemical hypertrophy, NA	Mitochondrial KATP channel, mitochondrial membrane potential, oxidant status, total antioxidant status, superoxide dismutase	(Guven, 2018)
Porcine cardiac progenitor cells	2D Cell culture, Chemical hypertrophy, Ang II, ET1	MiR-21, MiR-29a, GATA4, and MEF2c Expression	(Zlabinger et al., 2019)

Ang II, Angiotensin II; PE, Phenylephrine; Iso, Isoproterenol; ET1, Endothelin-1; NA, Noradrenaline.

When considering which hypertrophy model to choose for a study, various factors need to be considered beforehand. The most prominent factor in research is often time and cost effectiveness, which acts in favor of 2D chemically stimulated cultures, however this is not the best option in quality of results in some cases. The analysis of certain genes, proteins and non-coding RNAs might be insufficient in chemical stimulated cell hypertrophy models, as these do not trigger all the pathways that would be activated *in vivo*, which can lead to a false read-out and interpretations (Carreño et al., 2006). 3D models, such as organoids, can be stimulated mechanically and show a better comparability to *in vivo* conditions. These also allow to investigate the crosstalk between different cell types, especially the three main types within the heart cardiomyocytes, fibroblast, and endothelial cells, which are often not considered in regular 2D models. Fibroblasts especially play an important role in fibrosis and remodeling during hypertrophy processes and are therefore important to investigate (Travers et al., 2016). Organoids appear as the most ideal option in regard to simulating hypertrophy conditions *in vivo*, while still considering the principles of 3R (replacement, reduction, and refinement). Improving and also simplifying organoid technology should be the goal in translational research, so it can be used more widely for disease modeling such as cardiac hypertrophy instead of animal models.

## New Pathways in Cardiac Hypertrophy Involving Non-Coding RNAs

Among some well-known and established pathways in cardiac hypertrophy new signaling cascades have been identified over the last few years, especially with the rise of non-coding RNAs. Non-coding RNAs include amongst others miRNAs, lncRNAs, and circRNA. Some have been shown to play a role in remodeling and hypertrophy induction, either in promoting or inhibiting the process (Wehbe et al., 2019). These non-coding RNAs have also been investigated as potential treatment targets in cardiac hypertrophy, which may lead to new therapeutic approach for patients in the future (Li et al., 2018).

### miRNAs Influence Hypertrophy Pathways

MiRNAs are small non-coding RNAs with a length of approximately 22 nucleotides and interfere with mRNAs through complementary binding causing degradation or inhibition of transcription (Fabian and Sonenberg, 2012; Wehbe et al., 2019).

#### Pro-Hypertrophic miRNAs

Recently, Zhang et al. showed that miRNA-29 played a significant role, as overexpression seemed to inhibit angiotensin II induced LV hypertrophy in a mouse model. Collagen I and III secretion and TGFβ and pSMAD2/3 levels were downregulated indicating that hypertrophy was indeed hindered (Zhang et al., 2020). MiRNA-26a also seems to show anti-hypertrophic effects in rat models and in cell culture, as it targets the 3’-UTR of the GATA4 mRNA (Liu et al., 2016). Similar effects on remodeling can be seen with miRNA-101, as an overexpression in a rat hypertrophy model showed reduced gene expression and protein levels of known hypertrophy related genes like Rab1a, ANF, and b-MHC and reduced relative cell areas (Wei et al., 2015). MiRNA-133 downregulation has been found to aid hypertrophy development, however restoring the expression during hypertrophy induction only reduced fibrosis and apoptosis, but not hypertrophy signaling itself (Abdellatif, 2010).

#### Anti-Hypertrophic miRNAs

On the other hand, miRNAs can also induce or help induce hypertrophy, as shown with, e.g., miRNA-22. MiRNA-22 overexpression has been shown to be able to induce hypertrophy *in vitro* by modulating PTEN levels without adding any other stimulating agent and further showed to be crucial for phenylephrine and angiotensin II hypertrophy induction (Xu et al., 2012; Huang et al., 2013). MiRNA-217 similarly promotes cardiac hypertrophy interfering with PTEN levels. Additionally, catalysis of histone 3 lysine 9 dimethylation (H3K9me2) and mRNA downregulation of euchromatic histone–lysine N-methyltransferases (EHMT1/2) have been described to imitate fetal heart associated conditions further leading to hypertrophy development (Inagawa et al., 2013; Thienpont et al., 2017). MiR-155 is another player that has been identified to promote cardiac hypertrophy, by causing an inflammatory response with macrophage migration and signaling (Heymans et al., 2013). Additionally, miRNA-200c has been identified to interfere in MAPK/ERK pathway upregulation by targeting dual-specific phosphatase-1 (DUSP-1) (Singh et al., 2017).

### LncRNAs and circRNAs: Importance in Remodeling Pathways

LncRNAs can regulate promotors, enhancers and insulators by conformation changes and forming secondary structures further influencing expression of other genes. Moreover, lncRNAs can modulate miRNAs by acting as a sponge and therefore influence genes post-transcriptionally. The lncRNA myosin heavy‐chain‐associated RNA transcripts (MHRT) has been described to influence hypertrophy induction, as it antagonizes chromatin‐remodeling factor Brg1, which further activates β‐MHC. MHRT is encoded in the same gene locus (beta-cardiac muscle myosin heavy chain gene) as miRNA-208, which promotes cardiac hypertrophy (Callis et al., 2009; Han et al., 2011; Han et al., 2014). Another prominent lncRNA, identified by Wang et al. (2014a), is cardiac hypertrophy related factor (CHRF). The authors showed that CHRF was upregulated in cardiac hypertrophy and heart failure. CHRF acts as a sponge for a miRNA‐489, therefore de‐represses the myeloid differentiation primary response gene 88 (Myd88) and subsequently induces remodeling (Wang et al., 2014a). Cardiac‐apoptosis‐related lncRNA (CARL) has also been shown be strongly expressed in hypertrophy remodeling by inhibiting miR‐539 (Muthusamy et al., 2014; Wang et al., 2014b).

CircRNAs are circularized RNAs which are covalently closed. They are more stable than linear RNAs due to this structural feature and have recently gained an increasing research attention. However, they have not been as excessively studied as miRNAs and lncRNAs, leaving room for further studies on circRNAs in hypertrophy remodeling and in cardiovascular diseases in general (Hansen et al., 2013; Ottaviani and Martins, 2017; Li et al., 2018). CircRNAs can, similarly to lncRNAs, act as a sponge for miRNAs, thereby inhibiting them. CircRNA ciRS-7/CDR1as has been identified as a sponge for miR-7, which has been shown to be upregulated in patients with left ventricular hypertrophy (Hansen et al., 2013; Kaneto et al., 2017). The heart-related circRNA (HRCR) has also been found to inhibit miRNA-233 in the same way, therefore de-repressing the activity-regulated cytoskeleton-associated protein (K. Wang et al., 2016).

CircRNAs and LncRNAs provide a new approach for treatment due to their sponging properties and they may present a new biomarker for predicting the severity of the remodeling process and in heart failure. However, some challenges for non-coding RNAs as therapeutic option must still be faced, including off-target effects and delivery (Dong et al., 2018).

## Conclusions and Outlook

Cardiac hypertrophy will remain an ever-present clinical challenge due to several widespread risk factors in an increasingly aging patient population. Advanced and refined tissue engineering approaches give an opportunity to study pathological pathways extensively without requiring *in vivo* animal studies, which are more costly, time- and labor-intensive. These approaches allow the identification of possible biomarkers and the testing of therapies in a setting that mimics *in vivo* conditions more closely, although not perfectly as some factors like immune interactions are still lacking. The induction of cardiac hypertrophy *in vitro* is predominantly performed with pharmacological agents, such as angiotensin II, as mechanical stress induction poses a greater challenge.

Several well-known pathways which are involved in hypertrophic remodeling have been identified, including newer additions mostly revolving around non-coding RNAs like miRNAs, lncRNAs, and circRNAs. Non-coding RNAs that aid or negatively interfere with cardiac hypertrophy have been identified by inhibiting other factors (supporting or inhibitory) in the hypertrophy signaling cascades. There have been dozens of studies on a great number of different factors involved in this complex pathological process. Further, also epigenetic modifications have been discovered to be regulated and influenced to some degree by non-coding RNAs, which are involved in cardiac hypertrophy (Dong et al., 2018). However, it is important to further investigate and develop even more advanced tissue engineering options to allow a more profound understanding of remodeling pathways and subsequently the development of new pharmacological targets and treatment approaches.

## Author Contributions

NK and KZ wrote the main body of the manuscript. AS, JM-T, EH, and DT assisted with literature research and comments regarding language and style. AS and MG revised the manuscript and assisted with structural conceptualization.

## Conflict of Interest

The authors declare that the research was conducted in the absence of any commercial or financial relationships that could be construed as a potential conflict of interest.
